# A Label-Free DNA-Immunosensor Based on Aminated rGO Electrode for the Quantification of DNA Methylation

**DOI:** 10.3390/nano11040985

**Published:** 2021-04-12

**Authors:** Mina Safarzadeh, Ahmed Suhail, Jagriti Sethi, Anas Sattar, David Jenkins, Genhua Pan

**Affiliations:** 1 Wolfson Nanomaterials and Devices Laboratory, School of Engineering, Computing and Mathematics, Faculty of Science and Engineering, University of Plymouth, Devon PL4 8AA, UK; ahmed.suhail@plymouth.ac.uk (A.S.); jagriti.sethi@plymouth.ac.uk (J.S.); D.F.Jenkins@plymouth.ac.uk (D.J.); gpan@plymouth.ac.uk (G.P.); 2 School of Biomedical and Healthcare Sciences, Peninsula Schools of Medicine and Dentistry, University of Plymouth, Devon PL4 8AA, UK; anas.sattar@plymouth.ac.uk

**Keywords:** reduced graphene oxide (rGO), quantification of DNA methylation, amination, NH_2_ chemisorption, MGMT gene

## Abstract

In this work, we developed a sandwich DNA-immunosensor for quantification of the methylated tumour suppressor gene O-6-methylguanine-DNA methyltransferase (MGMT), which is a potential biomarker for brain tumours and breast cancer. The biosensor is based on aminated reduced graphene oxide electrode, which is achieved by ammonium hydroxide chemisorption and anti-5-methylcytosine (anti-5mC) as a methylation bioreceptor. The target single-strand (ss) MGMT oligonucleotide is first recognised by its hybridisation with complementary DNA to form double-stranded (ds) MGMT, which is then captured by anti-5mC on the electrode surface due to the presence of methylation. Raman spectroscopy, X-ray photoelectron spectroscopy (XPS) and Scanning electron microscopy (SEM) techniques were used to characterise the electrode surface. Cyclic voltammetry (CV) and differential pulse voltammetry (DPV) techniques were used for electrochemical measurements. Under optimised conditions, the proposed biosensor is able to quantify a linear range of concentrations of the MGMT gene from 50 fM to 100 pM with a limit of detection (LOD) of 12 fM. The sandwich design facilitates the simultaneous recognition and quantification of DNA methylation, and the amination significantly improves the sensitivity of the biosensor. This biosensor is label-, bisulfite- and PCR-free and has a simple design for cost-efficient production. It can also be tailor-made to detect other methylated genes, which makes it a promising detection platform for DNA methylation-related disease diagnosis and prognosis.

## 1. Introduction

DNA methylation is an epigenetic modification (alterations in gene expressions without changing the sequence) of DNA that plays an important role in regulating cellular function. It has been shown that changes in DNA methylation patterns may be associated with various diseases including cancer [1,2]. DNA methylation is the covalent binding of a methyl group (–CH${}_{3}$) to the 5th carbon atom of a cytosine nucleotide that follows a guanine nucleotide (CpG sites) [2]. There are different techniques to detect DNA methylation. Conventional techniques based on molecular biology including bisulfite treatment, methylation-specific PCR (MSP), mass spectrometry (MS) and liquid chromatography (LC) have been used extensively [3,4,5]. These techniques are occasionally used together and may rely on each other. Despite advantages such as high sensitivity and not being affected by DNA imperfections, these techniques require expensive equipment, large amounts of samples and specific expertise and are limited by analysis time, which are disadvantages when detecting DNA methylation. In recent years, considerable effort has been directed towards the development of biosensors and DNA sequencing techniques that have the potential to overcome the limitations of the aforementioned techniques, along with portability and amenability to miniaturisation [3]. Biosensors can either be used to detect DNA methylation on their own or can be combined with conventional techniques [6].

Povedano et al. [7] reported two different electrochemical affinity biosensors to detect methylated DNA. In the first method, anti-5-methylcytosine (anti-5mC) was used to capture methylated DNA followed by another antibody conjugated with peroxidase used as the detector element. In the second method, a capture probe was immobilised on the surface and was used to hybridise the DNA. After hybridisation, the methyl group was captured by anti-5mC and a secondary antibody conjugated with peroxidase was used as the detector element. In the former technique (the immunosensor), the biological reactions took place on the surface of carboxylic acid-modified magnetic beads (HOOC-MBs), while in the latter (the DNA sensor), they are placed on Streptavidin-modified MBs (Strep-MBs). In both of the techniques, MBs were then magnetically captured on a screen-printed electrode followed by an amperometric detection of the target gene. The limit of detection (LOD) was reported to be 6.8 pM for the immunosensor with relative standard deviation (RSD) = 3.9% and 42 pM for the DNA sensor with RSD = 4.8%. Chen et al. [8] reported an electrochemical stem-loop-tetrahedron composite DNA-probe platform immobilised on a AuNP-coated gold electrode. After immobilisation of the composite DNA probe on the electrode surface, complementary DNA was added and hybridisation took place. Streptavidin-horseradish peroxidase (S-HRP) and an appropriate substrate were used to amplify the signal. This platform showed a broad dynamic range of 1 aM to 1 pM, and the LOD was 0.93 aM. Khodaei et al. [9] developed an immunosensor using reduced graphene oxide (rGO) and anti-5mC to capture methylated DNA, which was then hybridised with ssDNA-conjugated Fe${}_{3}$O${}_{4}$ nanoparticles. The LOD for this biosensor was reported to be $9\times 10^{-5}$ ng/mL (0.6 fM). Daneshpour et al. [10] developed a chip format sandwich biosensor for the analysis of DNA methylation using Fe3O4/N-trimethyl chitosan/gold (Fe3O4/TMC/Au) nanocomposite as the label. In this work, polythiophene (PT) was used as an immobilisation platform for antibodies. The linear range of concentration for this biosensor was reported to be 50 fM to 5 nM, and the LOD was 2 fM.

Graphene has beneficial electrical, mechanical and optical properties. It has high mobility for charge carriers, high electrical conductivity and large surface area (2630 m${}^{2}$/g) [11,12]. Moreover, the electrochemical performance of graphene and its derivatives such as graphene oxide (GO) and reduced graphene oxide (rGO) are shown to be higher compared to other electrodes such as glassy carbon (GC), graphite and carbon nano tubes (CNTs) [12]. The electron transfer behaviour of graphene shows well-defined redox peaks using cyclic voltammetry (CV) in redox active solutions such as [Fe(CN)${}_{6}$]${}^{3-/4-}$ and [Ru(NH${}_{3}$)${}_{6}$]${}^{3+/2+}$. Additionally, the apparent electron transfer constant (k${}^{0}$) is higher in graphene than GC, indicative of a faster electron transfer [13]. The aforementioned properties along with the presence of defects, disorders and functional groups on the surface of graphene, GO and rGO makes them suitable for biosensing platforms [11]. Defects provide active sites for electron transfer and oxygen-containing functional groups to help in oxidation reactions by reducing the overpotential voltage [14,15].

Li et al. [16] developed a novel graphene–rGO double layer biosensor for the detection of a DNA antigen. The reported electrochemical biosensor is label-free and requires no signal enhancement and complicated immobilisation. The biosensor showed a linear range from 10${}^{-10}$ to 10${}^{-7}$ M and a limit of detection of 1.58 × 10${}^{-13}$ M (0.158 pM). He et al. [17] fabricated an electrochemical biosensor based on amine functionalised rGO–Fe${}_{2}$O${}_{3}$ nanocomposite modified glassy carbon electrode (GCE). This biosensor was used for the detection of rutin, and CV and second derivative linear sweep voltammetry (SDLSV) were used to investigate the sensor performance. The linear range was reported to be 6.0 nM to 80 μM with a LOD of 4 nM. Haque et al. [18] reported a DNA–graphene affinity biosensor for the detection of regional DNA methylation in a collection of DNA samples taken from cancer cell lines as well as cancer tissues. Their method is based on the adsorption affinity of graphene-modified electrodes towards DNA nuclebases (guanine (G) > adenine (A) > thyamine (T) > cytosine (C)). The reported biosensor is able to distinguish fully, partially and non-methylated DNA sequences with single CpG resolution and requires no sequencing analysis.

This paper introduces an immuno-DNA-based electrochemical biosensor for label-free detection of the MGMT tumor suppressor gene. In this biosensor, reduced graphene oxide electrodes (rGO) modified with anti-5-methylcytosine antibody (anti-5mC) were used to capture the antigen. A novel technique is introduced to functionalise the rGO surface with nitrogen-containing functional groups. Functionalisation of amine groups was achieved by chemisorption of ammonium hydroxide at the oxygenated and defect sites of the rGO surface. Raman and XPS techniques confirmed the formation of a layer of N-containing functional groups, with the amine groups as the dominant group (amination). Amination facilitates antibody immobilisation, allowing femtomolar concentration to be detected. This approach can potentially be used to detect any methylated gene that is known as a disease biomarker. It can be beneficial in point-of-care (POC) programs as an inexpensive tool because signal enhancing and target labeling are not required.

## 2. Materials and Methods

### 2.1. Reagents and Solutions

All of the reagents used in this study were of analytical grade. Tris-EDTA (TE) pH 8.0, phosphate buffered saline (PBS) pH 7.2, bovine serum albumin (BSA), sodium chloride (NaCl), potassium ferricyanide (K${}_{3}$[Fe(CN)${}_{6}$]), potassium chloride (KCl) and ammonium hydroxide solution 28% (NH${}_{3}$(aq)) were purchased from Sigma-Aldrich (UK). Recombinant protein G was obtained from ThermoFisher (UK). PBS tablets, pH 7.4, were purchased from Fisher Scientific (UK), and the PBS buffer solution was prepared in Milli-Q water. Mouse anti-5-methylcytosine monoclonal antibody (anti-5mC) was purchased from Zymo research (USA). All the synthetic nucleic acids were obtained from Integrated DNA Technologies (USA). The purchased single-stranded (ss) DNA sequence of the MGMT oligonucleotide was GTCC $C_{M}$ GA $C_{M}$ GCC $C_{M}$ GCAG GTCCT $C_{M}$ GCGGTGCGCACCGTTTGCGACTTGGTG, where $C_{M}$ was methylcytosine. The complementary sequence was CACCAAGTCGCAAACGGTGCGCACCGCGAGGACCTGCGGGCGTCGGGAC.

### 2.2. Apparatus and Measurements

All of the electrochemical measurements were performed using a μStat ECL BiPotentiostat/Galvanostat purchased from Dropsens (Spain). The screen-printed electrodes (DRP-110RGPHOX) were also obtained from Dropsens. The electrodes had rGO as the working electrode, carbon as the counter electrode and silver as the reference electrode.

Electrochemical measurements (cyclic voltammetry, CV and differential pulse voltammetry, DPV)were carried out for the bare electrode and after each incubation step. The measurements were performed in 100 μL of 10 mM PBS pH 7.4 solution containing 10 mM K${}_{3}$[Fe(CN)${}_{6}$] and 1M KCl as electrolyte agents. CV scans were obtained over a potential range of 0.55 and −0.2 V using a scan rate of 100 mV/S. DPV scans were performed over a potential range of 0.45 and −0.15 V, a pulse duration of 40 ms and a scan rate of 100 mV/s. All of the measurements were carried out at room temperature.

Raman spectra were obtained using a XPLORA HORIBA system combined with an Olympus BX41 microscope. A 532 nm green laser source with a power of 100 mW, 100× objective lens, a scan range of 1100 to 3000 cm${}^{-1}$ and an exposure time of 5–60 s were used to characterise the electrodes.

A Thermo Scientific Nexsa X-Ray Photoelectron Spectrometer System was used to carry out XPS analysis using a monochromatic Al Kα X-ray source (1486.68 eV). The pass energy for wide scans was 200 eV, with an energy step size of 1 eV and 10 scans. The pass energy for high resolution scans was 40 eV, with an energy step size of 0.1 eV and 20 scans.

**Figure A1:**
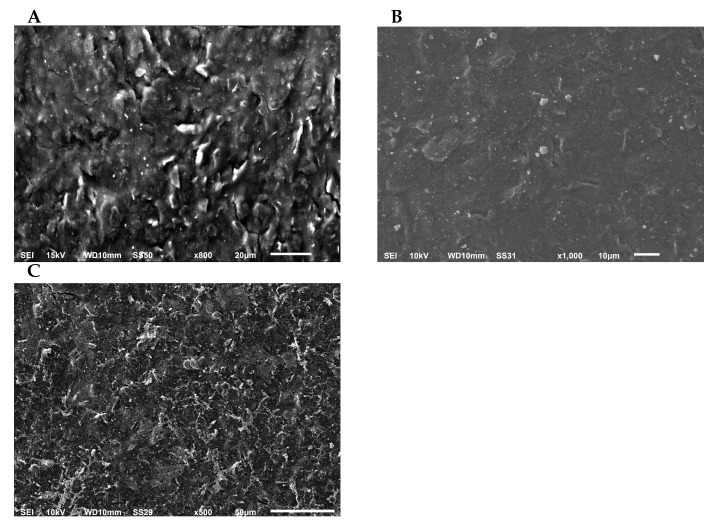
SEM images of a bare rGO electrode (**A**), an aminated rGO electrode (**B**) and aminated electrodes after incubation in the antibody (**C**).

Scanning electron microscopy (SEM) was performed using a JEOL 6610LV SEM. The SEM images of the rGO electrode and the electrode after incubation in ammonium hydroxide and antibody are provided in the Appendix A (Figure A1).

### 2.3. Preparation Steps

In order to immobilise the functional groups that facilitate immobilisation of antibodies on the surface, the rGO electrodes were first incubated in ammonium hydroxide solution (28.0–30.0% NH${}_{3}$ basis) for 2 h at room temperature. Subsequently, the aminated electrodes were dried with nitrogen and were kept in a vacuum for further use.

At the time of experiment, the aminated electrodes were first incubated in a mixture of anti-5mC and protein G (70:30) both diluted in PBS pH 7.2. Protein G is a bacterial membrane protein that is commonly used for immobilisation of oriented antibodies [19]. Protein G is known for its affinity to the non-antigenic (Fc) regions of antibodies, leaving the antigen binding sites available to bind to their target antigen [20,21,22]. After immobilisation of antibody and protein G mixture, the unbound functional groups were blocked using 1% BSA in PBS pH 7.4. Finally the sensor was incubated in different concentrations of ssDNA and hybridised target MGMT oligonucleotides. The electrode was washed with 300 μL of PBS pH 7.4 after each incubation step to remove unbound molecules and after each measurement to clean the surface and prepare for the next incubation step.

The hybridisation of ssDNA strands was performed as follows: a single-stranded MGMT target and its complementary strand were first brought to a concentration of 1 μM using TE buffer containing 50 mM of NaCl (TE-NaCl). Then, 200 μL of each was added to a vial and the mixture was heated at 65 ${}^{\circ }$C for five minutes to facilitate hybridisation. The mixture was then diluted with TE–NaCl and kept at 4 ${}^{\circ }$C for short term use.

**Figure A2:**
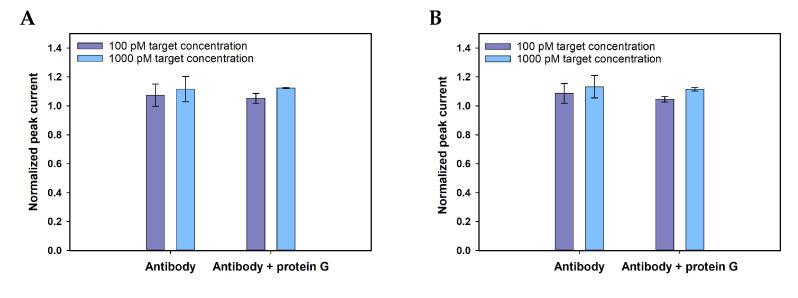
Detection of 100 (purple) and 1000 (blue) pM of the target gene using either the antibody or antibody conjugated to protein G, using both CV (**A**) and DPV (**B**) techniques.

**Figure A3:**
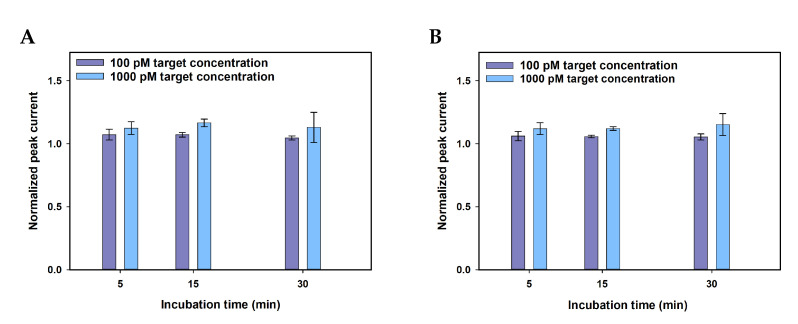
Detection of 100 (purple) and 1000 (blue) pM of the target gene with various bovine serum albumin (BSA) optimisation times (5, 15 and 30 min), using both CV (**A**) and DPV (**B**) techniques.

**Figure A4:**
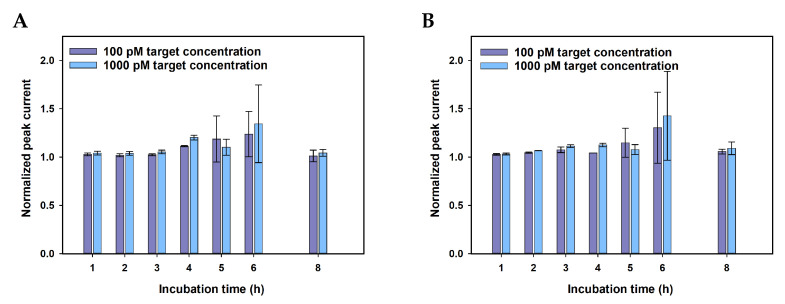
Detection of 100 (purple) and 1000 (blue) pM of the target gene of the target gene with various antibody incubation times (1, 2, 3, 4, 5, 6 and 8 h) using both CV (**A**) and DPV (**B**) techniques.

**Figure A5:**
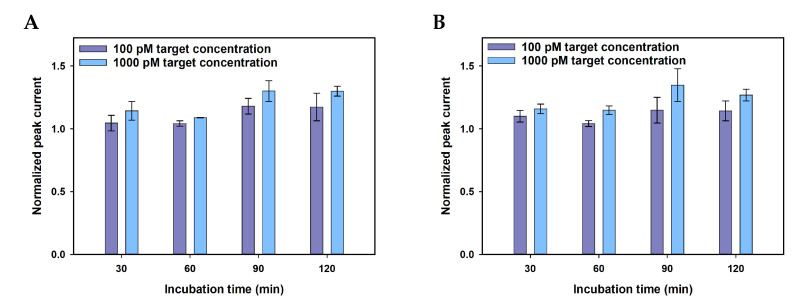
Detection of 100 (purple) and 1000 (blue) pM of the target gene of the target gene with various antigen incubation times (30, 60, 90 and 120 min) using both CV (**A**) and DPV (**B**) techniques.

Various experimental variables involved in the biosensor preparation were optimised to achieve the best possible sensitivity and LOD. This includes the optimisation of antibody, BSA and antigen incubation times as well as application of protein G (with or without protein G). A detailed description is provided in the Appendix A (Figure A2, Figure A3, Figure A4 and Figure A5). The evaluated variables, the tested ranges and the optimal values are summarised in Table A1. The optimal conditions were used to perform the linear regression and selectivity studies. A scan rate experiment was also performed, and the results are provided in Figure A7.

## 3. Results and Discussion

### 3.1. Characterisation of the Sensing Electrode

The rGO SPEs were incubated in ammonium hydroxide solution (28.0–30.0% NH${}_{3}$ basis) for 2 h in order to functionalise amine groups on the surface, facilitating binding of the antibodies to the rGO surface (see Section 2.3). The presence of N-containing functional groups were confirmed using Raman and XPS.

#### 3.1.1. Raman

**Figure 1:**
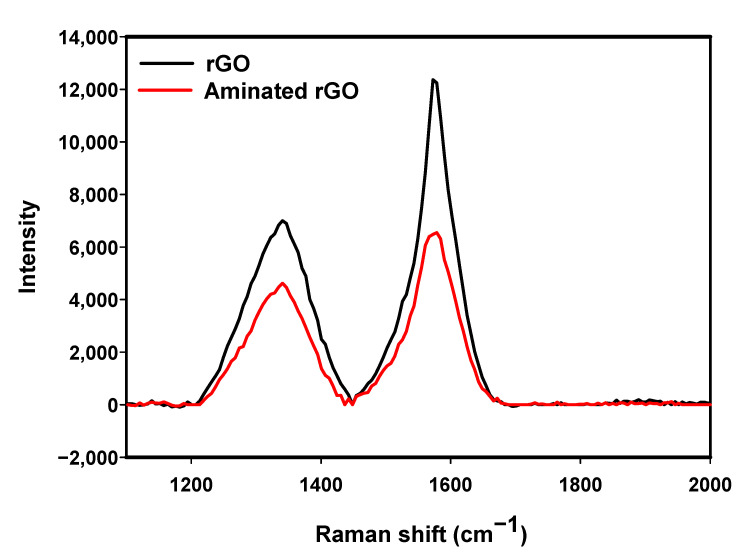
Raman spectra obtained from a bare reduced graphene oxide (rGO) electrode and rGO electrode incubated in ammonium hydroxide. Both the G (1578 cm${}^{-1}$) and D (1340 cm${}^{-1}$) bands decreased and broadened after rGO amination while the $I_{D}/I_{G}$ ratio increased from 0.6 to 0.7.

Raman spectra of the bare rGO electrode and the aminated electrode are compared in Figure 1. The strong peak at around 1578 cm${}^{-1}$ represents the in-plane vibrations of sp${}^{2}$-bonded graphitic carbon atoms (G band), while the weak peak at 1340 cm${}^{-1}$ is attributed to the out-of-plane vibration of disordered structures (D band) [23]. No shifts were seen in any of the bands after amination, which means no doping or strain occurred in the aminated rGO lattice [24,25,26]. In general, the peak intensity ratio ($I_{D}/I_{G}$) can be used to evaluate the ratio of structural defects or disorder level in the rGO layers [23]. The $I_{D}/I_{G}$ for the bare electrode was 0.6 while it increased to 0.7 for the aminated electrode suggesting the presence of more defect sites in the aminated rGO sample. These results are consistent with the results from Wei et al. [27] and Baldovino et al. [28]. Moreover, the full width at half maximum (FWHM) of the G peak of the Raman spectra is often used along with the $I_{D}/I_{G}$ ratio and gives further information about the level of defects of the sample [29]. The FWHM of the G peak increased from 60.5 to 78.2 after the amination of rGO samples, which confirms the presence of N atoms on the surface [28].

#### 3.1.2. XPS

**Figure 2:**
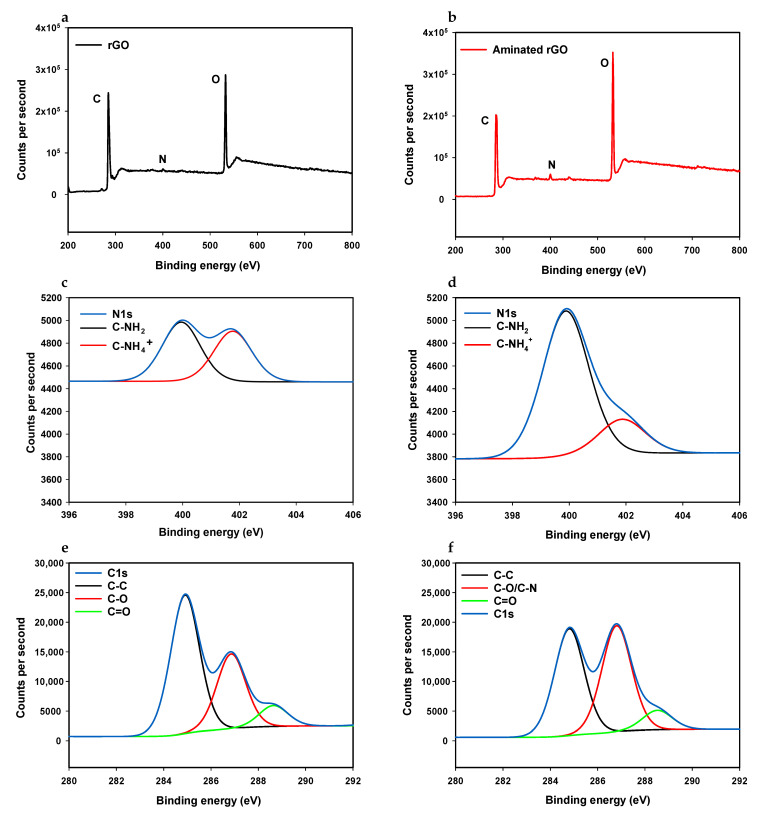
XPS spectra of rGO and aminated rGO electrodes. Survey scan of a bare rGO electrode (**a**) and an aminated electrode (**b**). N1s high resolution spectra of bare rGO (**c**) and aminated rGO (**d**) electrodes and C1s high resolution spectra of bare rGO (**e**) and aminated rGO (**f**) electrodes.

XPS measurements were carried out to quantitatively characterise the chemical composition of the bare and aminated rGO electrodes. Survey scans of the bare and the aminated rGO as well as C1s and N1s high-resolution scans of these samples are shown in Figure 2. As can be seen in Figure 2b, a nitrogen peak was observed at 400 eV in the survey scan of the aminated rGO, which confirms the presence of nitrogen-bearing molecules on the surface. However, a small N peak in the spectrum of bare rGO (Figure 2a) was also seen, which is due to the reduction process of graphene oxide [16]. The atomic ratio of nitrogen was calculated to be 2.09% for aminated rGO, while it was only 0.61% for the bare rGO sample. The integrated peak areas of N and C were used to calculate the N/C ratio for both samples. The N/C ratio was 3.07% for the aminated sample and 0.83% for the bare sample.

The N1s spectra showed asymmetrical profiles (Figure 2c,d). These can each be deconvoluted into two components. The peak located at 399.9 eV can be attributed to C-NH${}_{2}$, and the one located at 401.8 eV can be assigned to C-NH${{}_{4}}^{+}$ (quaternary nitrogen) [30,31,32]. Comparing the N1s spectra before and after amination showed that the C-NH${}_{2}$ peak height doubled while the C-NH${{}_{4}}^{+}$ peak height showed no significant change. This shows that C-NH${{}_{4}}^{+}$ was already embedded in the rGO lattice during the reduction process while the amination process only resulted in increasing the amount of NH${}_{2}$ on the surface (see Section 3.2).

The high resolution C1s spectra also exhibited asymmetrical shapes and tailing peaks for both aminated rGO and bare rGO samples (Figure 2c,f, respectively). They can each be deconvoluted into three component peaks. The main component peaks correspond to the presence of C atoms in C–C bonds (sp${}^{3}$ bonded carbons), located at 284.80 eV for the rGO sample and at 284.90 eV for the aminated sample [33]. The other component peaks are located at 286.81 and 286.86 eV for bare and aminated samples, respectively, which can be attributed to C–O or C–N bonds [30,34]. Finally, the peaks at higher binding energies, 288.53 and 288.64 eV, for bare and aminated electrodes, respectively, can be assigned to C=O bonds [35].

### 3.2. Ammonium Hydroxide Chemisorption

**Figure 3:**
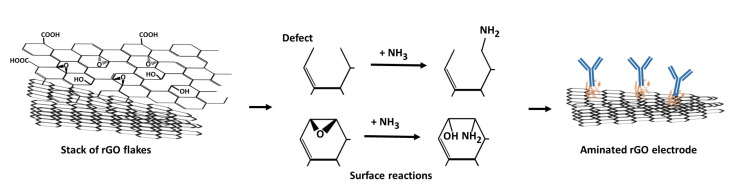
Schematic of the possible surface reactions that may occur on the rGO electrode after incubation in ammonium hydroxide. These reactions would lead to the presence of amine functional groups on the surface.

Nitrogen atoms can, in principle, be incorporated onto the surface in three ways. It can be either by replacing a carbon atom present in the lattice with an N atom, by replacing an existing functional group by an N containing functional group [28,36] or by binding to the defect sites and broken C–C bonds present in the rGO flakes [37,38]. Because of the absence of high temperature and pressure, in this study, it is assumed that the direct replacement of carbon by nitrogen atoms does not occur. Instead, NH${}_{3}$ is assumed to react with either defects in the lattice or with acidic sites of rGO that are oxygenated functional groups, namely, hydroxyl, epoxide and carboxyl groups [28]. Because the rGO SPE is made of stacked rGO flakes, many defect/vacancy sites are available for the attachment of amine groups (Figure 3). The presence of defects constitute instabilities of the structure of rGO, making these sites prone to bonding with ammonia to stabilise the structure [39]. In the case of reactions with oxygenated functional groups, due to the lack of high temperatures and suitable activators (e.g., thionyl chloride, carbodiimide or tosyl chloride), the only feasible reaction is that ammonia attacks epoxide groups as a nucleophile (Figure 3). In this reaction, a new bond with the carbocation adjacent to the epoxide group will form, resulting in epoxide opening, dissociation of NH${}_{3}$ and formation of an amino alcohol [38]. Our XPS experiments confirm that this is the dominant amination process, as shown by the increase in the C-NH${}_{2}$ component in the N1s high resolution spectra and the increase in the C–O/C–N component in the C1s spectra while the peak of C=O remained the same (Figure 2f). The above surface reactions do not affect the structure of rGO, as evidenced by the Raman spectra (Figure 1), which show that no major defects were introduced after amination.

Besides acting as a linker for antibody, these amine groups can form hydrogen bonds with adjacent or newly formed oxygen bearing groups such as OH⋯N and O⋯HN, which facilitate electron transfer by acting as electron donors to rGO and stabilise the structure [37,40].

### 3.3. Electrochemical Experiments

#### 3.3.1. Electrochemical Behaviour and Selectivity

**Figure 4:**
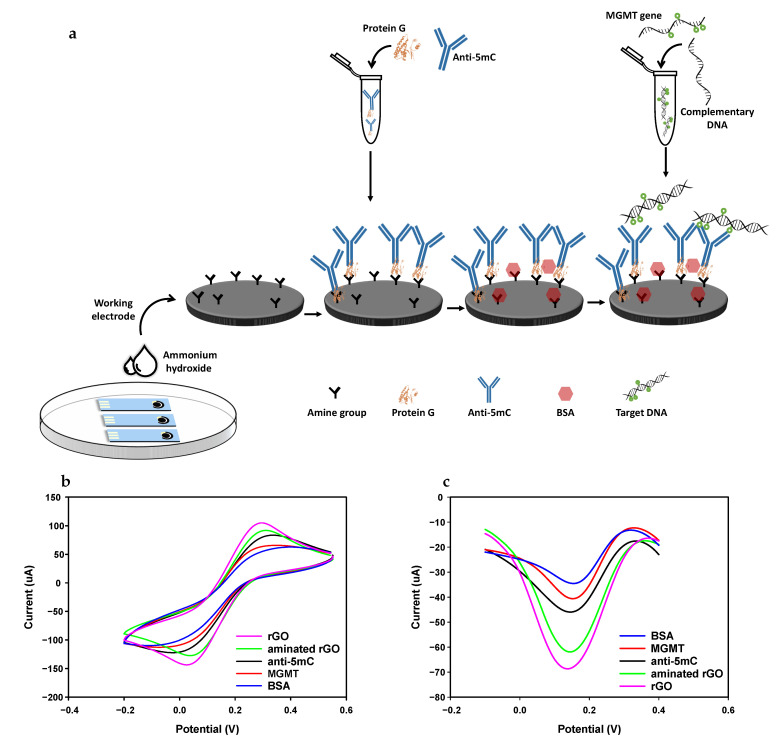
Schematic display of the developed method for the quantification of O-6-methylguanine-DNA methyltransferase (MGMT) oligonucleotide (**a**). Electrodes were incubated in ammonium hydroxide and were kept in a vacuum for further use. Cyclic voltammetry (CV) (**b**) and differential pulse voltammetry (DPV) (**c**) characteristics of the sensor after each assembly steps in 10 mM K${}_{3}$[Fe(CN)${}_{6}$] containing 1 M KCl.

After each incubation step, the electrochemical response was evaluated via the behaviour of voltammograms following structural changes in the biofunctional surface of the electrode. Figure 4a shows a schematic of the various preparation steps of the biosensor as well as measured voltammograms for the bare electrode (rGO), aminated electrode, antibody, BSA as the blocking agent and antigen for both CV (Figure 4b) and DPV (Figure 4c) measurement techniques at a scan rate of 100 mV/s.

As observed from the CV voltammograms, the anodic peak current (i${}_{pa}$) of the bare electrode is at 106.5 μA, which is due to the electrical conductivity and electron mobility of rGO and the available electroactive sites on the surface, which facilitate electron transfer [16,41]. After treating the electrode surface with ammonium hydroxide, the peak current decreased to 91.7 μA, which is due to the presence of new functional groups on the surface. The peak current further decreases to 83.4 μA after immobilisation of antibodies on the surface, which reduces the number of active sites for electron transfer. The peak current decreased once more to 55.8 μA after immobilising BSA on the surface, which is expected as it acts as an inert layer and blocks the surface, impeding electron transfer [42,43]. Finally, when the biosensor is incubated in 100 pM of the target dsDNA, the peak current rises to 65.6 μA, which can be attributed to ionic conductance and π–π interaction of the DNA duplex, which leads to increased charge transfer [44]. Although the peak current increased slightly after DNA incubation, it was still smaller than bare, aminated rGO and antibody peak currents. Additionally, after immobilisation in the antibody, a slight positive shift in the peak potential was observed, which is consistent with spatial blockage of the surface and impeded electron transfer [45]. The cathodic peak currents (i${}_{pc}$) of the CV voltammograms showed the same trend as the anodic peak current after each incubation step, with a corresponding negative shift in the cathodic peak current.

The DPV voltammograms for the various preparation and detection steps were in agreement with the CV voltammograms. The peak current of the voltammogram for the bare electrode was 71.7 μA. It decreased to 61.8 μA after incubation in ammonium hydroxide, followed by another decrease to 45.9 μA after immobilising the antibody on the surface. The peak current once more decreased to 34.5 μA after the sensor was incubated in BSA, and finally, it grew to 40 μA due to the presence of the target gene and the formation of immunocomplexes. In addition, there was a positive shift in the peak potential of the DPV voltammograms, which is due to the impeded electron transfer.

**Figure A6:**
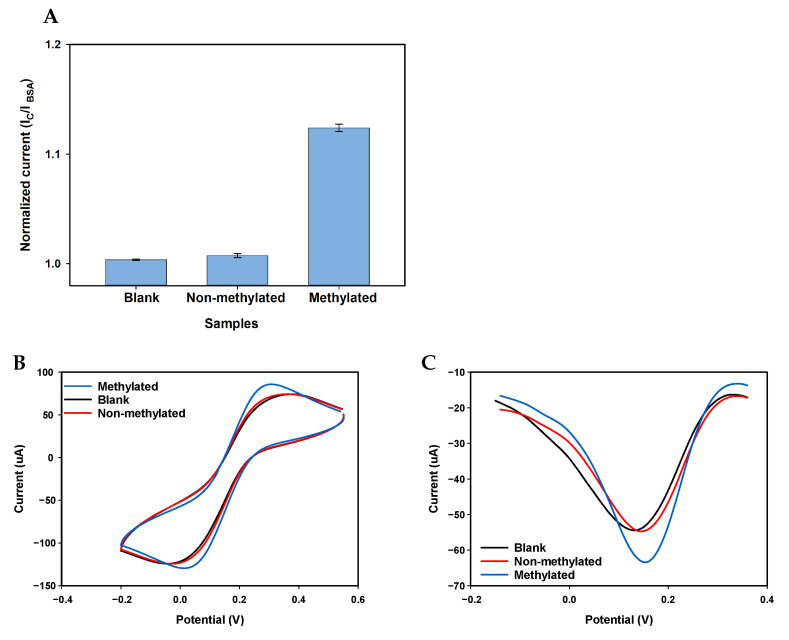
Comparison of the responses obtained from various targets (methylated, non-methylated and blank) using the proposed biosensor. Normalized CV peak current for different samples measured on three different replicas (**A**). CV (**B**) and DPV (**C**) voltammograms of one replica for each sample.

**Figure A7:**
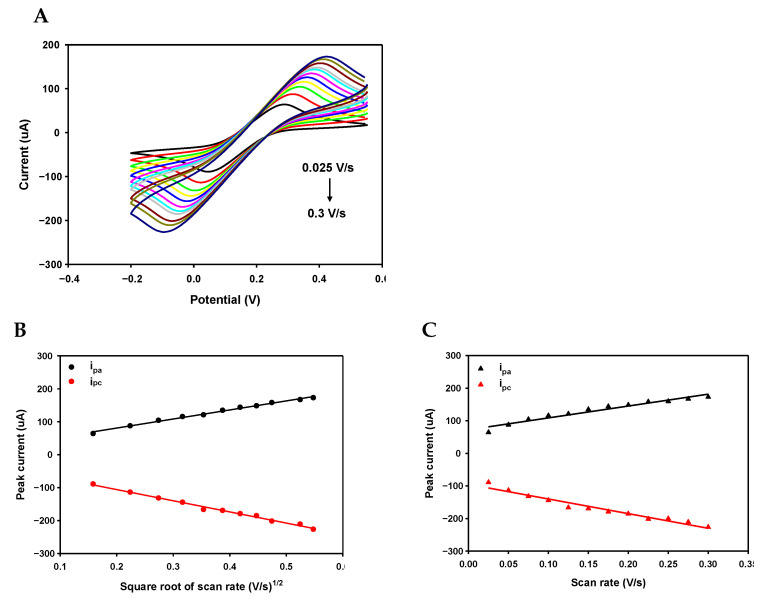
CV voltammograms of the aminated electrode under various scan rates from 0.025 V/s to 0.3 V/s (0.025, 0.05, 0.075, 0.1, 0.125, 0.15, 0.175, 0.2, 0.225, 0.275 and 0.3 V/s) (**A**). Anodic and Cathodic peaks as a function of the square root of the scan rate (**B**) and the scan rate (**C**).

Selectivity tests were performed using methylated DNA, non-methylated DNA and a blank sample. The results are provided in the Appendix A (Figure A6). All of the above measurements were carried out under the same measurement conditions.

#### 3.3.2. Biosensor Linearity

**Figure 5:**
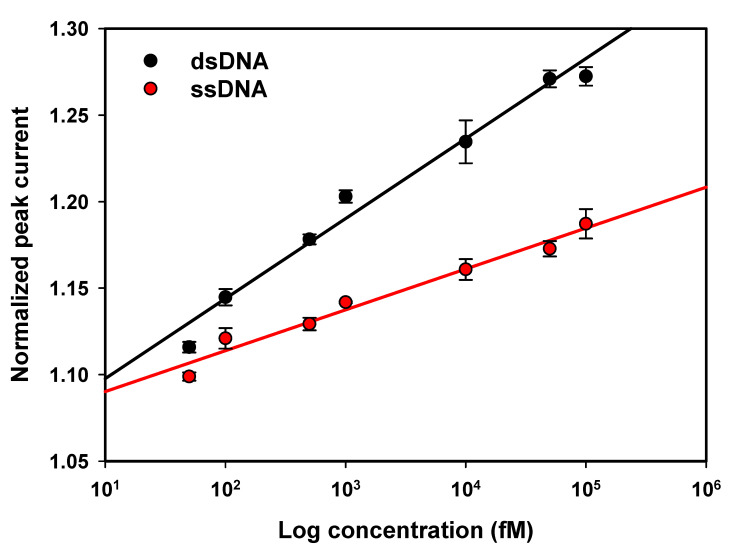
Calibration curves constructed with normalised peak currents of DPV responses as a function of the logarithm of the concentration of target ssDNA (red) and dsDNA (black). For both targets, the current increases with increases in concentration. Error bars are the standard deviation of three replicates.

DPV measurements were performed for biosensors incubated in various concentrations of single-stranded and double-stranded MGMT oligonucleotides. The results are shown in Figure 5, where the normalised peak currents are plotted as a function of the logarithm of the concentration. The normalised peak current increases with an increase in the concentration, which is in agreement with the results reported by Povedano et al. [46] for the MGMT gene.

Best fit linear models and their corresponding R${}^{2}$ values are as follows:

ssMGMT: y=0.0092ln(x)+1.0767 with $R^{2}=0.9893$

dsMGMT: y=0.0184ln(x)+1.0665 with $R^{2}=0.991$

The LOD was calculated using the equation LOD=3.3σ/m, where σ is the standard deviation of the DPV response of 16 blank samples and *m* is the slope of the calibration curve. For ssDNA, the LOD was 25 fM, and for dsDNA, it was calculated to be 12 fM. To date, to the best of our knowledge, only two groups reported better LODs for the detection of DNA methylation, but these groups used nanoparticles (Table A2).

Importantly, these results indicate that the response of the biosensor is different for single-stranded DNA compared to double-stranded DNA. This is due to the fact that ssDNA forms a coiled shape while dsDNA makes a stretched helix shape, allowing electrons to be conducted through the duplex and taking advantage of the π-stacks in the duplex [47,48,49].

Due to the usage of hybridisation, the biosensor is selective to the target oligonucleotide when applied to dsDNA, but it is not selective when detecting ssDNA because it responds to the methyl group regardless of the ssDNA sequence. However, the different responses of the sensor to dsDNA versus ssDNA can be used to achieve selectivity when applied to a sample with an unknown mixture of methylated single-stranded oligonucleotides. When hybridising the unknown mixture using the complementary target and diluting the sample to obtain various concentrations, the formation of dsDNA (or lack thereof) can be measured.

## 4. Conclusions

A novel sandwich immuno-DNA biosensor was developed for label-free, rapid and sensitive detection of MGMT oligonucleotides. The biosensor is based on commercially available screen-printed reduced graphene oxide (rGO) electrodes that were aminated using ammonium hydroxide solution. Amination was performed in order to provide amine functional groups on the surface acting as a linker to immobilise biomolecules. Raman results suggested the presence of nitrogen atoms on the surface, which were further confirmed to be C-NH${}_{2}$ groups via XPS. Electrochemical detection of the MGMT oligonucleotide was achieved by hybridising the single-stranded synthetic oligonucleotide with its complementary sequence and by capturing the methylation with an antibody. Under optimal conditions, the biosensor showed a LOD of 12 fM for a double-stranded MGMT gene without any PCR amplification, bisulfite treatment or labelling. The reproducibility and stability of the sensor over time still needs to be explored in the future. Additionally, the electrochemical performance of the aminated rGO may still be improved using elevated temperatures and pressures during ammonium hydroxide incubation. Finally, the response of the proposed technique in plasma samples needs to be tested in future studies. The proposed technique can be modified to detect other methylated target genes. This assay can form the basis for clinical applications in diagnostics and patient monitoring due to its ability to rapidly detect epigenetic biomarkers, high sensitivity and simplicity.

## Data Availability

The data that support the findings of this study are available up on reasonable request from the corresponding author.

## Appendix A

### Appendix A.1. SEM

Scanning electron microscopy (SEM) was used to characterise the surface of the bare rGO electrode, the aminated electrode and the aminated electrode after incubation in the antibody. These SEM images are presented in Figure A1. Figure A1a shows the surface of a bare rGO electrode. The image shows surface irregularities of 5–10 μm. In comparison, Figure A1b shows an aminated rGO electrode with a smoother surface. Figure A1c shows the successful immobilisation of biomolecules on the surface.

### Appendix A.2. Optimisation

Various experimental variables were optimised for the purpose of seeking the highest possible sensitivity of the biosensor. High sensitivity is critical in the quantification of methylation of the target oligonucleotide due to its presence in only a small subset of cells in a clinical specimen [50,51]. The relevant experimental steps that were optimised are antibody, BSA and antigen incubation times as well as the presence of protein G. The evaluated variables, their ranges and the optimal values are summarised in Table A1. In all of the optimisation tests, the CV and DPV responses of 100 and 1000 pM of target MGMT oligonucleotides were evaluated to select the optimal conditions. The error bars in all of the experiments are the standard deviation of three replicates.

**Table A1:** Optimised preparation steps and selected values.

Experimental Variable	Range	Selected Value
Application of protein G	With-without protein G	With protein G
Anti-5mC incubation time, hours	1–8	4
BSA incubation time, hours	5–30	15
MGMT gene incubation time, hours	30–120	60

The first optimisation step was understanding the effect of protein G functionality. For this, the sensor was incubated with the antibody conjugated and without being conjugated to protein G. As shown in Figure A2, using protein G decreased the error bars compared to not using protein G and the target concentrations, leading to enhancement in distinguishability for the biosensors as the error bars of both concentrations do not overlap. These results are due to the fact that the presence of protein G acts to preferentially align the antibodies on the electrode surface, resulting in an increased antigen-binding capacity of the antibody (Elshafey et al. [19]).

BSA incubation time was optimised by incubating three groups of electrodes in BSA for either 5, 15 or 30 min. The results (Figure A3) showed that, among these three, an incubation time of 15 min was optimal, as the error bars are the smallest and the increase in the normalised peak current between 100 and 1000 pM in both techniques are consistent. In contrast, the normalised peak currents for both 15 and 30 min of the BSA incubation time showed larger error bars, which can be due to the remaining unbound functional groups at the shorter 5 min incubation time, and conversely, saturation of the electrode surface with BSA molecules for the longer 30 min incubation time. Therefore, the incubation time of 15 min for BSA was selected as the incubation time in further studies.

The antibody incubation time was optimised by incubation of the electrodes in the antibody for 1, 2, 3, 4, 5, 6 or 8 h. Figure A4 shows that the optimal incubation time was 4 h, resulting in very small, non-overlapping error bars between 100 and 1000 pM. Additionally, the normalised peak current shift as well as the error bars were consistent in both measurement techniques in all incubation times. However, in shorter incubation times (1, 2 and 3 h), there was a small shift between 100 and 1000 pM, which shows that the sensor is unable to distinguish between these two concentrations. Additionally, for longer incubation times (5, 6 and 8 h), the error bars are larger, meaning that the result of the biosensor is not reproducible. Hence, 4 h incubation time was selected as the optimised antibody incubation time.

In order to optimise the antigen incubation time, the sensors were incubated with the antigen for 30, 60, 90 or 120 min. The results for this experiment are shown in Figure A5. These results show that, although there was a shift between 100 and 1000 pM in all of the groups, the error bars were large and overlapped in almost all of the cases except for the group that was incubated in antigen for 60 min. Consequently, 60 min was chosen as the optimised incubation time for the antigen.

### Appendix A.3. Selectivity

In order to investigate the selectivity of the proposed biosensor protocol, the biosensors were incubated in blank samples as well as 100 pM of single-stranded methylated and non-methylated targets and the response was measured with both the CV and DPV techniques. The blank sample was the buffer and the non-methylated oligonucleotide had the same sequence as the methylated MGMT oligonucleotide, with no methylation sites. The results (Figure A6) show that there is a significant difference after incubation in methylated DNA while no such difference is observed after incubation of the sensor in blank and non-methylated DNA. This test therefore shows that the proposed biosensor is selective towards the methyl groups on the methylated gene.

### Appendix A.4. Scan Rate Experiment

The scan rate experiments of an aminated rGO electrode were performed in PBS pH 7.0 containing 10 mM [Fe(CN)${}_{6}$] and 1 M KCl in order to study the redox reactions on the surface. Figure A7a shows the effect of varying the scan rate (0.025 to 0.3 V/s) on the voltammograms of the aminated rGO electrode, where the magnitudes of both the anodic ($E_{pa}$) and cathodic ($E_{pc}$) peak currents increase linearly with the increase in the square root of the scan rate (Figure A7b). This suggests that the electrochemical reaction is a diffusion-controlled process [44]. The diffusion coefficient (D) of the redox couple from the electrolyte to the aminated electrode was calculated using Randles–Sevcik equation [52]:

(A1) $$I_{p}=\left(2.69\times 10^{5}\right)n^{3/2}AD^{1/2}Cv^{1/2}$$

where $I_{p}$ is the peak current of the aminated electrode (in A), *n* is the number of electrons involved (*n* = 1), *A* is the surface area of the electrode (in cm${}^{2}$, *A* = 0.126 cm${}^{2}$), *D* is the diffusion coefficient, *C* is the concentration of the redox species (in mM, *C* = 10 mM) and *v* is the scan rate (in V/s, *v* = 0.1 V/s, which was used throughout the paper). *D* is therefore calculated to be 1.17 × 10${}^{-6}$ cm${}^{2}$/s.

The anodic and the cathodic peak potentials as well as the peak to peak separation (potential peak shifts, $\Delta E_{p}$ = $E_{pa}$−$E_{pc}$) increase with the scan rate (Figure A7c). Redox couples in which the peak to peak separations show a linear relationship with the scan rate are categorised as quasi-reversible reactions [53], and it is an indication of facile charge transfer kinetics in the scan rate range [44]. Therefore, the aminated electrodes provide adequate accessibility to electrons in transferring between the antibody and the aminated electrode. The effective heterogeneous electron transfer rate constant ($K_{s}$) was calculated using the Lavrion model [44]:

(A2) $$K_{s}=mnFv/RT$$

where *m* is the peak to peak separation (in V, *m* = 0.362 V), *n* is the number of electrons involved (*n* = 1), *F* is the Faraday constant (96,485 J/mol), *v* is the scan rate (in V/s, *v* = 0.1 V/s), *R* is the gas constant (8.314 J/mol.K) and *T* is room temperature (298 K). The $K_{s}$ value was calculated to be 1.41 s${}^{-1}$ at 0.1 V/s, which indicates fast electron transfer between the amine groups and the rGO electrode.

#### Appendix A.4.1. Comparison with Other Works

The various parameters of the proposed biosensor are compared with other electrochemical affinity techniques reported so far for the detection of DNA methylation in Table A2.

**Table A2:** An overview of the electrochemical affinity biosensor to date for the detection of DNA methylation.

Electrode	Bioreceptor	Dynamic Range/LOD	Technique	Reference
Gold modified with gold nanoparticles	DNA probe, methyl binding protein, gold nanoparticle, antibody	0.5–500 pM/0.17 pM	DPV	[54]
SPCE modified with polythiophene	Anti-5mC and Fe${}_{3}$O${}_{4}$/N-trimethylchitosan/gold nanocomposite	0.01–1000 pM/0.002 pM	DPV	[10]
SPCE	Biotinylated DNA probe immobilised on magnetic beads and anti-5mC	87–2500 pM/26 pM	DPV	[46]
SPCE	Anti-5mC immobilised on magnetic beads and biotinylated DNA probe	3.9–500 pM/1.2 pM	DPV	[7]
SPCE modified with rGO and polyvinyl alcohol	Anti-5mC immobilised and DNA probe conjugated with Fe${}_{3}$O${}_{4}$-citric acid nanocomposites	7×10${}^{-4}$–140.29 pM/6.31× 10${}^{-4}$ pM	DPV/EIS	[9]
rGO modified with ammuniom hydroxide	Anti5-mC and complementary DNA	0.5–100 pM/0.012 pM	DPV/EIS	This work
